# Rett syndrome

**DOI:** 10.4103/0019-5545.55959

**Published:** 2005

**Authors:** Prabhat Sitholey, Vivek Agarwal, Rohit Srivastava

**Affiliations:** * Professor and Head, Department of Psychiatry, K.G. Medical University, Lucknow 226003; ** Assistant Professor, Department of Psychiatry, K.G. Medical University, Lucknow 226003; *** Junior Resident, Department of Psychiatry, K.G. Medical University, Lucknow 226003

**Keywords:** Rett syndrome, neurodevelopmental disorder, pervasive developmental disorder

## Abstract

Rett syndrome is a rare, progressive, neurodevelopmental disorder that has been reported only in the girl child. We describe the case of a 6.9-year-old girl with Rett syndrome. She had normal development till the age of 2 years. However, over the next 4–5 months, she lost her acquired, purposeful hand skills; expressive and receptive language; and reciprocal social interaction; and gradually developed a broad-based gait and typical midline stereotyped hand movements (mouthing, rubbing).

## INTRODUCTION

Rett syndrome is a neurodevelopmental disorder that exclusively affects the girl child. It is characterized by normal development till 6 months to 2 years of age followed by gradual loss of purposeful hand movements and development of characteristic, stereotypical hand movements; loss of previously acquired speech; psychomotor retardation; ataxia; deceleration of head circumference; and autistic symptoms. The prevalence of this syndrome is estimated to be between 1 in 10,000 and 1 in 22,000.^1^ There are only three reports of Rett syndrome from India.^2^,^3^ We report a case from India of Rett syndrome to create more awareness about this syndrome.

## THE CASE

A 6.9-year-old girl presented with gradual loss of acquired speech, social interaction and hand skills, stereotyped movements of the hand and body, and faecal and urinary incontinence for the past 4.9 years.

## Birth and early developmental history

The patient's mother had a full-term, normal, vaginal delivery at home. The child had apparently normal motor, social and language development till 2 years of age.

## Clinical history

After two years of normal development, she started showing signs of developmental regression. Her mother noticed that gradually her vocabulary started decreasing and within 4–5 months, she lost all the words that she had learnt previously. She would neither speak any meaningful word nor compensate for this by non-verbal communication. The mother also noticed her lack of attachment with family members. She would not interact or make eye contact with them, even when the family members would try to do so. The mother noticed that she had lost her acquired, purposeful hand skills. She would no longer hold, pick up or grasp things in her hands. She developed stereotyped, midline hand movements. She would keep both her forearms flexed in front of the chest in a manner that one hand would be kept over or under the palm of the other hand and she would move or rub one hand over the other. She would rub or roll her thumb on the finger. The child would wet her hands with saliva, move her body from one side to another shifting her weight on either foot, walk with a broad-based gait and by putting down the toes before the heel. She would keep moving aimlessly in this manner.

She would no longer indicate her toilet needs, defaecate and urinate in her clothes and remain unconcerned. It was noticed that she would smile or cry without any apparent reason. She also developed an erratic sleep pattern. She would sleep for the initial 2–3 hours in the night and then get up and walk about aimlessly. At times, she would again go back to sleep. Her mother observed that she was unable to chew semisolid food and would swallow it. For the past 1 year, she had become irritable. She would scream, pull her hair, hit the mother or roll on the floor if somebody tried to stop her hand movements or, at times, would do so without any reason. She could care little for herself and would be uncooperative if someone tried to help her. The frequency of her crying episodes also increased. She developed generalized tonic–clonic seizures for the past 4 months.

There was no history of any significant medical or psychiatric illness in the past. There was a family history suggestive of mania in her father.

### Examination

On examination, her motor activity was found to be increased. There was no evidence of hyperventilation. She had characteristic midline stereotyped hand and body movements as described above. She did not establish eye contact with her mother or others, and showed no interest in social interaction. She showed no separation anxiety when her mother went outside the interview room. In the ward, she showed no interest in toys or in playing with other children. Her neurological assessment was within normal limits. Her head circumference was 45.5 cm (less than two standard deviations from the mean for age).^4^ On the Vineland Social Maturity Scale,^5^ her developmental age was 1 year and developmental quotient (DQ) 14.5. She was assessed using the Developmental Behavior Checklist,^6^ which is a screening questionnaire for autism with a cut-off score of 17; she scored 30.

### Investigation

Her haemogram was normal. No abnormality was found on CT scan and plain MRI of the head. An EEG revealed an interictal pattern of generalized seizure. On brain stem-evoked response audiometery (BERA), her left auditory pathway was intact indicating normal hearing in the left ear but on the right side, BERA was not recordable.

### Diagnosis

She was diagnosed as a case of Rett syndrome as per ICD-10 criteria.^7^ Important differential diagnoses considered were childhood autism, childhood disintegrative disorder, Landau–Kleffner syndrome, tuberous sclerosis, encephalitis and infantile neuronal ceroid lipofuscinosis (INCL). Unlike childhood autism and childhood disintegrative disorder, Rett syndrome exclusively affects girls and has distinct midline stereotyped hand movements and deceleration in head growth. In Landau–Kleffner syndrome, the onset is usually at 5–5.5 years whereas in Rett syndrome, the onset is usually between 6 and 24 months. Mental retardation is not found in patients with Landau–Kleffner syndrome and they usually have a myoclonic type of seizure. The absence of adenoma sebaceum and normal neuroimaging of the brain ruled out the possibility of tuberous sclerosis. INCL is similar to Rett syndrome in the initial stages, both being characterized by rapid psychomotor developmental regression and hand stereotypies. In addition, in INCL, there is a progressive loss of vision, which is not found in Rett syndrome. Absence of visual disturbances and absence of marked atrophy of the brain ruled out the diagnosis of INCL.^8^

### Management

On 5 mg/day of olanzapine her irritability decreased and sleep disturbance improved. In two weeks^1^ time, she stopped bed-wetting on 50 mg of night-time imipramine. Her mother was taught intensive speech stimulation and gross body movement imitation techniques. Seizures were controlled with 375 mg/day of valproate. The child was also regularly sent to a playroom to involve her in play activities.

## DISCUSSION

The diagnosis of Rett syndrome is based on careful clinical assessment. This child fulfilled all the diagnostic criteria for Rett syndrome. We cannot comment about the deceleration in head growth with confidence, as the head circumference measurement at birth was not available. However, on examination, the head circumference was found to be much smaller than expected for her age. In some MRI studies, diffuse atrophy has been noted (most notably in the prefrontal, posterior frontal and anterior temporal regions).^1^ But in this case, neuroimaging (CT scan head and plain MRI head) was normal. The finding of a normal BERA on the left side was helpful in ruling out the presence of hearing loss as the underlying cause of the loss in language skills.

The clinician should differentiate between mental retardation and Rett syndrome. In mental retardation, the child improves with time while in Rett syndrome, there is a gradual decline with time. All female children with low intelligence and autistic symptoms should be suspected of having Rett syndrome. Developmental stagnation; deceleration in head circumference; disinterest in the environment which starts between the ages of 6 and 12 months; developmental regression; loss of acquired hand skills, expressive and receptive language; and typical midline stereotypical movements between 1 and 3 years should alert the clinician to the possibility of Rett syndrome. This is a progressive disorder in which the patient becomes wheelchair-bound due to rigidity and dystonia, and develops scoliosis in the late stages leading to decreased longevity.

There is no specific treatment for Rett syndrome. Combined medical and psychosocial treatment in the form of occupational, physical and speech therapy is required to manage medical and behavioural problems. The child showed some improvement with this treatment and became more manageable.

Although the genetic analysis was not done in this case, the cause of Rett syndrome has recently been identified as mutation in the *MECP2* gene on chromosome Xq28.^9^ With this discovery, further research may lead to better understanding of the neurobiology of the disease and to specific treatment. It is important for clinicians to be aware of this disorder because increased identification will help in greater understanding of this disorder and proper guidance will help the patient and family, and reduce the burden of care on the parents.
